# Comparative Organellar Genomics of Pellidae: Insights into Codon Usage, Nucleotide Diversity, and Structural Evolution

**DOI:** 10.3390/plants15070997

**Published:** 2026-03-24

**Authors:** Wiktoria Czochór, Kamil Koczwara, Natan Pupek, Piotr Górski, Joanna Szablińska, Jakub Sawicki, Monika Szczecińska

**Affiliations:** 1 Department of Botany and Evolutionary Ecology, University of Warmia and Mazury in Olsztyn, Plac Łódzki 1, 10-719 Olsztyn, Poland; natanpopek@gmail.com (N.P.); joanna.szablinska@uwm.edu.pl (J.S.); 2 Department of Genetics and Plant Pathophysiology, University of Warmia and Mazury in Olsztyn, Plac Łódzki 3, 10-719 Olsztyn, Poland; kamil.koczwara@uwm.edu.pl (K.K.); jakub.sawicki@uwm.edu.pl (J.S.); 3 Department of Botany, Poznań University of Life Sciences, J.H. Dąbrowskiego 159, 60-594 Poznań, Poland; piotr.gorski@up.poznan.pl

**Keywords:** Pellidae, liverworts, *atp1* gene, organellar genomes, phylogenomics

## Abstract

Liverwort organellar genomes are generally highly conserved, but the subclass Pellidae (simple thalloids) shows unusual variation. This ancient yet unexplored lineage of simple thalloid liverworts provides an excellent model for investigating organellar genome evolution. In this study, we assembled four new plastid and four new mitochondrial Pellidae genomes using Oxford Nanopore sequencing, supplementing 86 plastomes and 82 mitogenomes from databases. We assessed nucleotide diversity and codon usage, and inferred phylogenies using IQ-TREE with fossil-calibrated dating. Plastomes ranged 120.6–126.5 kb, and mitogenomes 109–180 kb, with *Apopellia endiviifolia* featuring an exceptionally reduced mitogenome (~109 kb). Native RNA sequencing enabled a revised annotation of the mitochondrial *atp1* gene in *Apopellia*, revealing two introns (previously thought absent) and reducing the intergenic region share to 36.26%, the lowest known among liverworts. Comparative analyses revealed contrasting evolutionary dynamics between organelles: Plastomes displayed higher nucleotide diversity and phylogenetically inconsistent codon usage patterns, likely influenced by compositional bias, whereas mitogenomes were more conserved and largely consistent with established phylogenetic relationships among the orders. Phylogenomic analyses yielded discordant topologies: Chloroplast data recovered Pellidae as a monophyletic clade, whereas mitochondrial data placed Pelliales (*Pellia/Apopellia*) as basal Jungermanniopsida, rendering Pellidae paraphyletic. Within Pellidae-relevant clades, several major divergences were dated to the Carboniferous–Permian, but with systematic chloroplast–mitochondrial offsets. These results highlight recurrent organellar incongruence and the dynamic evolutionary history of Pellidae organellar genomes.

## 1. Introduction

The organellar genomes of liverworts (Marchantiophyta) are widely considered to be among the most stable in the plant kingdom, characterized by rare instances of gene loss and structural rearrangements [1,2]. Despite this general stability, which has been maintained across millions of years of evolution, not all liverwort lineages have been equally explored in the field of organellar genomics. The subclass Pellidae represents one of the major lineages within simple thalloid liverworts (Jungermanniopsida), comprising three orders: Pelliales, Fossombroniales, and Pallaviciniales [3,4]. Despite their morphological simplicity and ancient evolutionary origin, Pellidae exhibits remarkable diversity in organellar genome architecture, particularly evident in the extraordinary mitogenome size reduction recently discovered in *Apopellia* [5]. This genomic plasticity, combined with the prevalence of cryptic speciation within the group [6,7,8], makes Pellidae an ideal model system for understanding organellar genome evolution in early land plants.

Recent advances in liverwort organellar genomics have challenged the traditional paradigm of structural conservation in bryophyte genomes. While early studies suggested that liverwort organellar genomes are among the most stable in land plants [9,10], emerging evidence reveals significant variation within and between lineages [11,12]. In Pellidae, the mitogenomes of *Apopellia* were found to be the smallest among all known liverworts, at approximately 109 kb, achieved through dramatic reduction in intergenic spacers while retaining all introns [5]. This contrasts sharply with the more typical mitogenome sizes in sister genera, where *Pellia* mitogenomes range from 142 to 179 kb, which raises fundamental questions about the evolutionary pressures and mechanisms driving organellar genome size evolution in these ancient plant lineages.

The phylogenetic relationships within Pellidae have been contentious, with organellar and nuclear datasets sometimes yielding conflicting topologies [5,6,8,11,12]. While chloroplast data support Pellidae monophyly with high support values, mitochondrial datasets have suggested alternative relationships, with Pelliales appearing as the earliest diverging lineage within Jungermanniopsida rather than forming a clade with other Pellidae orders. These cyto-organellar discordances, likely resulting from incomplete lineage sorting, differential evolutionary rates, or ancient hybridization events [13], highlight the need for comprehensive comparative genomic analyses across all three orders. Recent phylotranscriptomic analyses have revealed that ancient hybridization may be more common in liverwort evolution than previously thought, potentially contributing to the observed phylogenetic incongruences [12].

Despite recent progress, significant gaps remain in our understanding of Pellidae organellar genome evolution. Previous studies have been limited by incomplete taxonomic sampling, with organellar genomes available for only a fraction of genera within each order. The recent expansion of liverwort mitogenome sampling to 47 accessions has revealed unexpected variation in genome size and intron content, even within the supposedly stable liverwort lineages [9]. However, complete organellar genomes are still lacking for several key genera, including multiple species of *Pallavicinia*, *Fossombronia*, and *Moerckia*, preventing comprehensive comparative analyses of genomic features across the subclass. Furthermore, the temporal framework of Pellidae diversification remains poorly resolved, hampering our understanding of how organellar genome evolution has proceeded in relation to major geological and climatic events.

The identification and characterization of hypervariable genomic regions represent another critical knowledge gap. While Paukszto et al. [5] identified hotspots of nucleotide diversity within *Pellia* and *Apopellia*, systematic comparisons across all three orders are needed to identify conserved versus rapidly evolving regions that could serve as phylogenetic markers at different taxonomic levels. Recent studies have shown that substitution rates in liverwort organellar genomes, while generally slower than in angiosperms, are not as low as previously thought and exhibit considerable variation among lineages [11,14]. Such comparative approaches could also reveal order-specific patterns of molecular evolution and potential genomic synapomorphies.

Estimating divergence times among liverwort lineages remains challenging due to the scarcity of reliable, deep-time fossil calibration points and the ancient nature of these evolutionary splits [15]. Recent studies using all available evidence suggest that complex thalloid liverworts started appearing very early, in the Late Silurian–Early Devonian period [16,17,18]. However, other large-scale genetic studies propose that the first groups of current liverworts separated so long ago that their genetic differences might be too old to accurately measure with molecular clock methods [12]. The quality of the fossil dates used for calibration significantly changes the estimated split times [19,20]. Therefore, it is crucial to carefully select how we use fossil dates and account for the uncertainty when estimating these molecular timelines.

The main objective of this study was to conduct a comprehensive comparative analysis of organellar genomes within the Pellidae subclass, enabling the exploration of their evolutionary dynamics, structural differences, and phylogenetic relationships. To obtain a reliable and comprehensive overview of this rarely studied group, our analyses were based on an extensive, combined dataset comprising a total of 90 chloroplast genomes and 86 mitochondrial genomes. The foundation of this dataset consisted of sequences deposited in public databases (86 plastomes and 82 mitogenomes). To ensure that the comparative analyses were fully robust and meaningful, we expanded the taxonomic sampling with newly sequenced genomes (both plastid and mitochondrial, derived from the same individuals) of four species: *Apopellia endiviifolia*, *Fossombronia wondraczekii*, *Moerckia blyttii*, and *Moerckia hibernica*. While some genomic data were already available for the genus *Apopellia*, public databases still exhibited significant gaps in the representation of the genera *Fossombronia* and *Moerckia*. The deliberate, targeted sequencing of exclusively these missing taxa allowed for a substantial increase in sampling density within these less-studied evolutionary lineages. Ultimately, this approach completed the research framework of the studied group without the unnecessary duplication of already available sequences.

This comprehensive dataset ultimately allowed us to fulfill the following specific objectives: (1) identifying the most variable genomic regions within each order and across the entire Pellidae subclass; (2) reconstructing robust phylogenetic trees to resolve conflicting evolutionary relationships and test for cyto-nuclear discordance; (3) estimating divergence times for major lineages using fossil calibrations; and (4) investigating patterns of organellar genome evolution, including size variation, structural rearrangements, and codon usage bias.

## 2. Results and Discussion

### 2.1. Organellar Genomes of Pellidae

The chloroplast genomes of the analyzed Pellidae species ranged from 115,878 bp in *Pellia neesiana* to 126,538 bp in *Moerckia hibernica*, within the size range of published plastomes [5,21,22,23] (Table S1). All chloroplast genomes showed the typical quadripartite structure and organizational features of liverwort chloroplast genomes. The mitochondrial genome ranged from 108,928 bp in *Apopellia endiviifolia* to 180,233 bp in *Moerckia hibernica* (Table S1). The mitochondrial genome of *A. endiviifolia* is one of the smallest known liverwort mitogenomes [11]. In liverworts, this mitogenome size reduction is not associated with gene or intron loss but results from shortened intergenic regions. Unlike most liverworts, where intergenic spacers constitute nearly half of the mitogenome length, their proportion in *Apopellia* has been reduced [11]. This reduction without changes to the coding portion distinguishes *Apopellia* from mitogenome miniaturization in parasitic plants [24].

#### 2.1.1. Characteristics of Chloroplast Genomes

**Figure 1:**
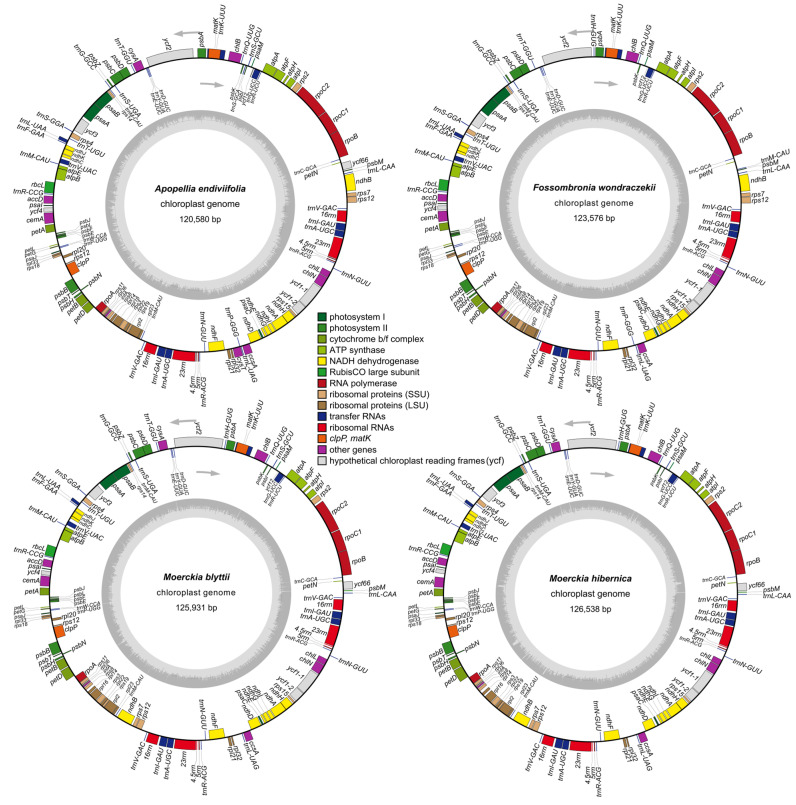
Chloroplast genomes of *Apopellia endiviifolia*, *Fossombronia wondraczekii*, *Moerckia blyttii* and *Moerckia hibernica*. Genes inside and outside the outer circle are transcribed in counterclockwise and clockwise direction, respectively. The genes are color-coded based on their function. The inner circle visualizes the GC content.

The newly sequenced plastid genomes are circular molecules containing regions typical of land plants (Figure 1). The plastomes ranged from 120,580 bp in *Apopellia endiviifolia* (*Apopellia endiviifolia 1*—PX421529) to 126,538 bp in *Moerckia hibernica* (PX421532) (Figure 1 and Table S1). The GC content varied: 35.9% in *A. endiviifolia*, 40.4% in *M. hibernica*, 41.3% in *Fossombronia wondraczekii* (PX421530), and 42.7% in *Moerckia blyttii* (PX421531). As previously published [5], 122 unique genes were identified in *A. endiviifolia*’s plastome: 81 protein-coding genes, four ribosomal RNAs, 31 transfer RNAs and six *ycf* genes of an indeterminate function (Figure 1 and Table S1). *F. wondraczekii* lacks *cysA* and *cysT* genes, and the *trnS(GCU)-psbI-trnS(GCA)* cluster (Figure 1).

**Figure 2:**
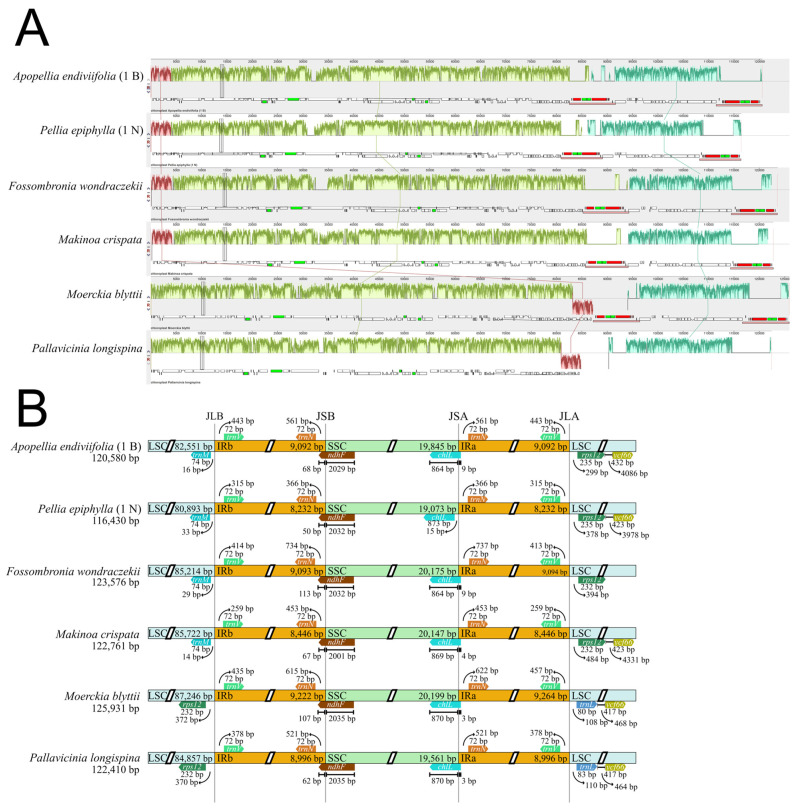
Comparative analysis of plastid genome structure and IR junctions in selected liverwort species (*Apopellia endiviifolia*, *Pellia epiphylla*, *Fossombronia wondraczekii*, *Makinoa crispata*, *Moerckia blyttii*, and *Pallavicinia longispina*). (**A**) Synteny alignment of the analyzed plastomes generated using -Mauve. Colored blocks represent homologous regions shared across the genomes, while shifts in block orientation indicate structural rearrangements. (**B**) Comparison of inverted repeat (IR) boundaries among the plastomes. Genes flanking the junction sites (JLB, JSB, JSA, and JLA) are indicated, along with their respective distances (in bp) from the corresponding borders.

Significant variation in gene placement was observed at the LSC/IR boundaries. In Pelliales and Fossombroniales, the JLB boundary is flanked by *trnM*, while *rps12* is at the JLA boundary. In contrast, Pallaviciniales showed translocation of the *rps12*–*rps7*–*ndhB* cluster, with *rps12* at JLB and *trnL* at JLA (Figure 2A and Figure S5). This rearrangement was previously reported in *Pallavicinia lyellii* [11].

The overall plastome architecture remained conserved among taxa, though gene positions relative to IR borders showed minor expansions and contractions (Figure 2B). Genes like *ndhF* (50–113 bp from JSB) and *chlL* (3–15 bp from JSA) showed slight positional variations.

In typical liverwort plastomes, *rps12* and *rps7* are located at the LSC region’s 5′ end. Deviations were found in *Conocephalum salebrosum*, where IR expansion created additional *rps12* and *rps7* copies, while *Conocephalum conicum* retained a typical structure [25].

#### 2.1.2. Characteristics of Mitochondrial Genomes

**Figure 3:**
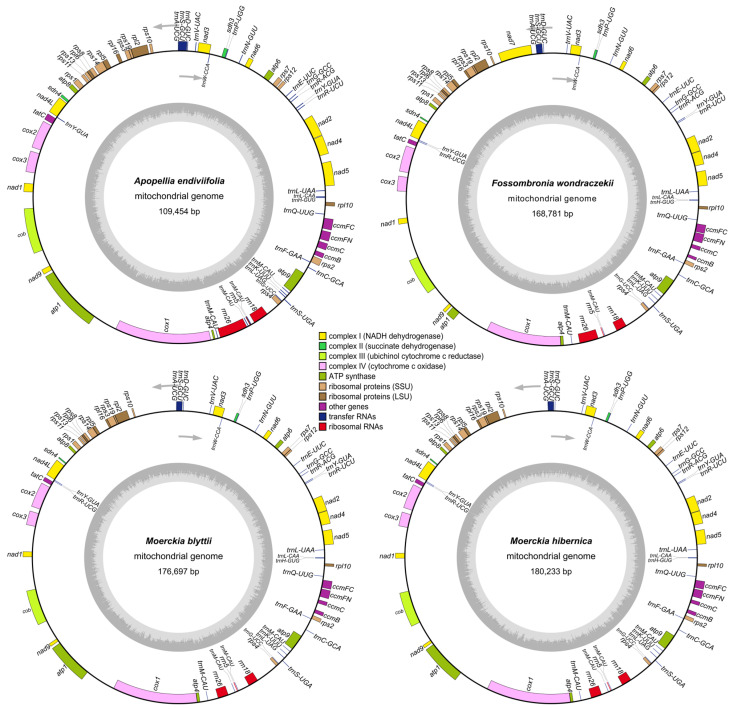
Mitochondrial genomes of *Apopellia endiviifolia*, *Fossombronia wondraczekii*, *Moerckia blyttii* and *Moerckia hibernica*. Genes inside and outside the outer circle are transcribed in counterclockwise and clockwise direction, respectively. The genes are color-coded based on their function. The inner circle visualizes the GC content.

The new mitochondrial genomes show nearly identical gene order and content, with 41 protein-coding genes, three ribosomal RNAs and 28 transfer RNAs, except for *trnR-UCG* loss in *A. endiviifolia* (Figure 3). Mitogenome sizes ranged from 109,454 bp in *A. endiviifolia* to 180,233 bp in *M. hibernica* (Figure 3 and Table S1). *F. wondraczekii* mitogenome lacks *atp1* gene introns, and two of nine *cox1* gene introns, while *A. endiviifolia* contains all introns despite being 60 kbp smaller. The reduction in *A. endiviifolia*’s mitogenome compared to other Pellidae is mainly due to nucleotide loss in intergenic regions and introns.

Analysis of *A. endiviifolia*’s compact mitogenome revealed important findings about the *atp1* gene structure. Long-read native RNA sequencing enabled an updated annotation of the *atp1* gene, showing two introns like Marchantiopsida, with 253 full-length transcripts mapped to the *nad9*-*cox1* region, including 229 supporting a tri-exonic structure (Supplementary Figure S5). Deep long-read sequencing confirmed heteroplasmy in coding and non-coding regions [23], with the new *atp1* annotation reducing the intergenic region share to 36.26%, the lowest among liverworts [11,26].

### 2.2. Codon Usage Differentiation of Pellidae Mitogenome and Plastome

**Figure 4:**
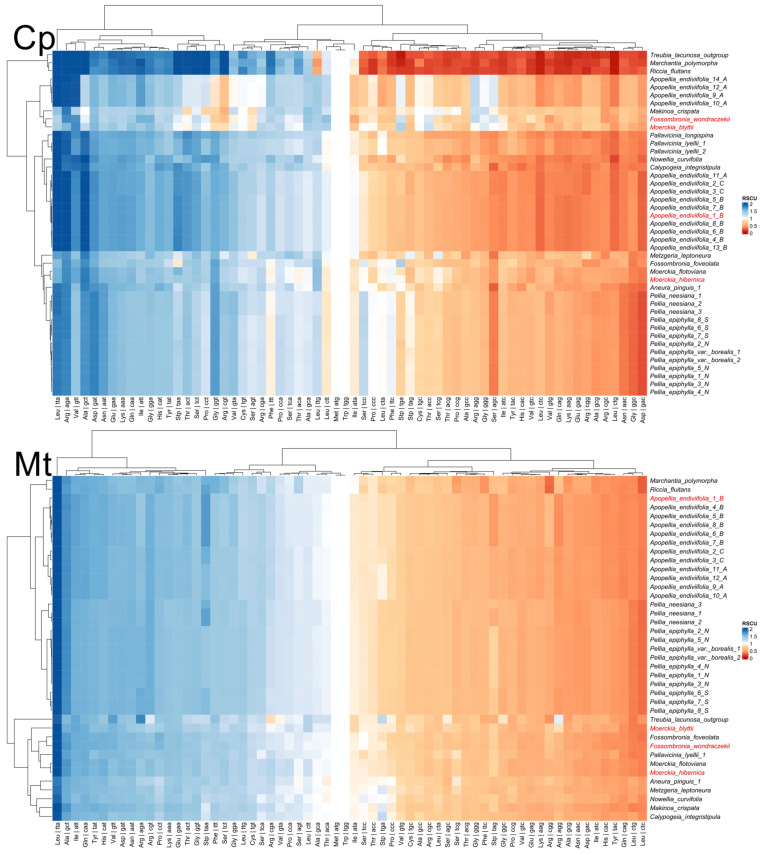
Codon usage of mitochondrial and chloroplast genomes for Pelliales, Pallaviciniales and Fossombroniales. Exact RSCU values are provided in Supplementary Materials (Tables S2 and S3).

RSCU analysis revealed distinct codon usage signatures at the ordinal level, differentiating major evolutionary lineages of Pellidae (Figure 4). To place the codon usage patterns of Pellidae in a broader phylogenetic context, representative liverwort taxa (*Treubia lacunosa*, *Marchantia polymorpha*, *Riccia fluitans*, *Novellia curvifolia*, *Metzgeria leptoneura*, and *Aneura pinguis*) were included in the RSCU analysis (Figure 4). These taxa represent major liverwort lineages and served as external references for comparison of plastid and mitochondrial codon usage patterns.

In plastomes, Pelliales (particularly *Pellia*) preferred TTA (Leu) and AGA (Arg) codons, while Fossombroniales and Pallaviciniales showed stronger signals for GCT (Ala) and GGT (Gly) (Figure 4). These shifts were reflected in stop codon usage: Pelliales relied almost exclusively on TAA, while TAG and TGA usage was more frequent in Fossombroniales and Pallaviciniales. Plastid RSCU patterns show inconsistencies with phylogeny, transgressing taxonomic boundaries, as evidenced by *Apopellia* partition into two clusters with divergent codon preferences. The grouping of one cluster with distant orders suggests systematic artifacts from plastome nucleotide composition.

Mitogenomes showed a more stable and taxonomically concordant signal at the ordinal level, with pronounced and homogeneous A/T bias, and clustering following established phylogenetic lines. Pallaviciniales showed conserved mitochondrial preference for ATT (Ile) and TCT (Ser), distinct from Pelliales. These patterns indicate plastid codon usage is more evolutionarily labile, while mitochondrial codon usage in Pellidae is more constrained and taxonomically concordant—which is important, because synonymous-site composition and codon usage bias generate systematic artifacts in phylogenomic inference if not modeled [26,27], and liverwort plastomes in particular often show U/A-ending preferences with contributions of mutation pressure and selection [28,29,30].

### 2.3. Nucleotide Diversity

**Figure 5:**
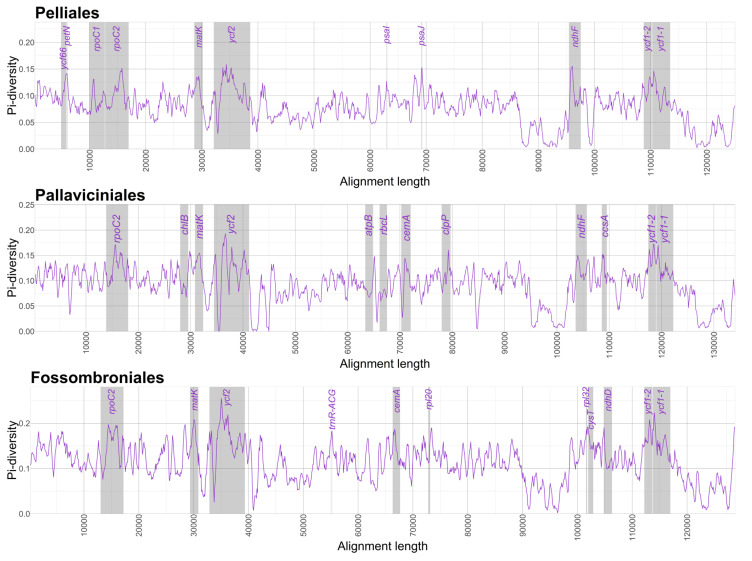
Comparison of nucleotide diversity between chloroplast genomes of Pelliales, Pallaviciniales and Fossombroniales with highlighted hotspots of pi-diversity. Sliding window analysis with windows size = 500 bp and window jump = 100 bp. π values are available in Supplementary Materials (Table S4); a separate alignment for each order can also be found in Supplementary Materials (Supplementary Files S1, S3 and S5).

Analysis of nucleotide diversity in plastid and mitochondrial genomes of the three analyzed orders of Pelliaceae (Pelliales, Pallaviciniales, and Fossombroniales) revealed similarities in diversity patterns, with π-diversity hotspots frequently occurring within the same genomic regions, while few hotspots were unique to individual orders (Figure 5). In plastid genomes, nucleotide diversity was unevenly distributed along the plastomes. Shared regions of elevated nucleotide diversity included: *ycf1-1*, *ycf2-2*, *ycf2*, *matK*, and *rpoC2*, with *ycf2* showing the highest π peak (π > 0.25) (Figure 5 and Table S4). Similar patterns were reported for *Pellia* and *Apopellia*, where nucleotide diversity was lower in inverted repeat (IR) regions than in single-copy regions (LSC and SSC), with major hotspots in *ycf1*, *ndhF*, and *rpoC2* [5]. The overlap of major hotspots (*ycf1*, *ycf2*, and *rpoC2*) suggests these loci maintain elevated substitution rates across deeper evolutionary divergences within Pellidae, while *ndhF* showed lineage-specific variability patterns (Figure 5). The high nucleotide diversity of *ycf1* and *ycf2* aligns with findings in *Aneura pinguis* cryptic species, where both genes were among the most polymorphic regions [31]. This pattern across species-, genus-, and order-level analyses highlights their utility for phylogenetic inference [32,33,34]. The *matK* gene was identified as a shared hotspot across all orders (π > 0.2) (Figure 5 and Table S4), supporting studies of *Aneura pinguis* and *Calypogeia*, where *matK* showed high nucleotide diversity and nonsynonymous SNPs [31,35].

**Figure 6:**
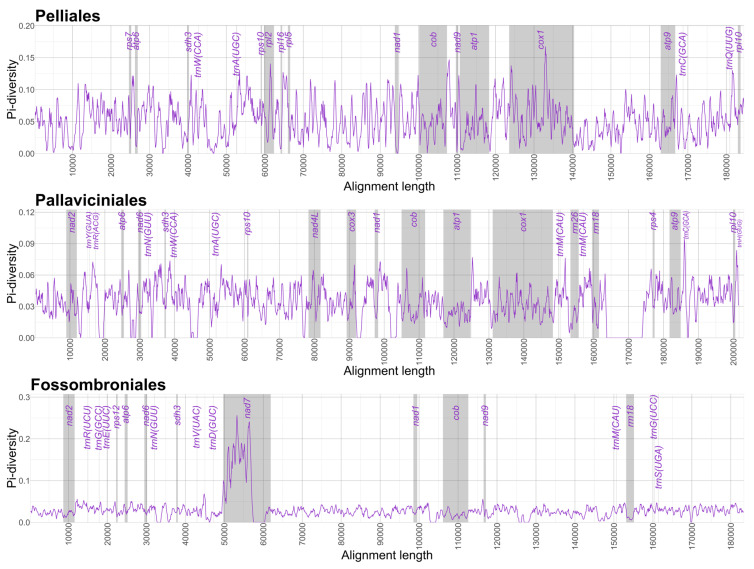
Comparison of nucleotide diversity between mitochondrial genomes of Pelliales, Pallaviciniales and Fossombroniales with highlighted hotspots of pi-diversity. Sliding window analysis with windows size = 500 bp and window jump = 100 bp. π values are available in Supplementary Materials (Table S4); a separate alignment for each order can also be found in Supplementary Materials (Supplementary Files S2, S4 and S6).

In contrast to plastid genomes, mitochondrial genomes of Pellidae showed lower nucleotide diversity, particularly in Pallaviciniales and Fossombroniales (Figure 6). In all orders, π values remained lower than in plastid genomes (Figure 5 and Figure 6), consistent with the conserved nature of liverwort mitochondrial genomes and contrasting with the dynamic mitochondrial genomes of vascular plants; this stability stems from low recombination rates, functional constraints, and nuclear surveillance [9,36]. The distribution of nucleotide diversity across mitochondrial genomes of the three Pellidae orders was characterized by low variability and limited hotspots (Figure 6). In Pelliales, protein-coding regions showed very low variation, with elevated π values mainly in non-coding regions, with only *cox1* showing elevated variability (π > 0.15) (Figure 6 and Table S4). In Pallaviciniales, nucleotide diversity remained low with few discrete peaks in intergenic spacers, with peaks in *cox2*, *cob*, and *cox1* (π > 0.06) (Figure 6 and Table S4). Fossombroniales showed the most homogeneous mitochondrial nucleotide diversity, with one pronounced hotspot in the *nad7* region, while the rest displayed minor π value fluctuations (Figure 6 and Table S4). These findings align with previous analyses showing low nucleotide diversity in protein-coding regions and concentrated variability in intergenic spacers and introns, with hotspots varying among related genera [5].

### 2.4. Phylogenetic Relationship

**Figure 7:**
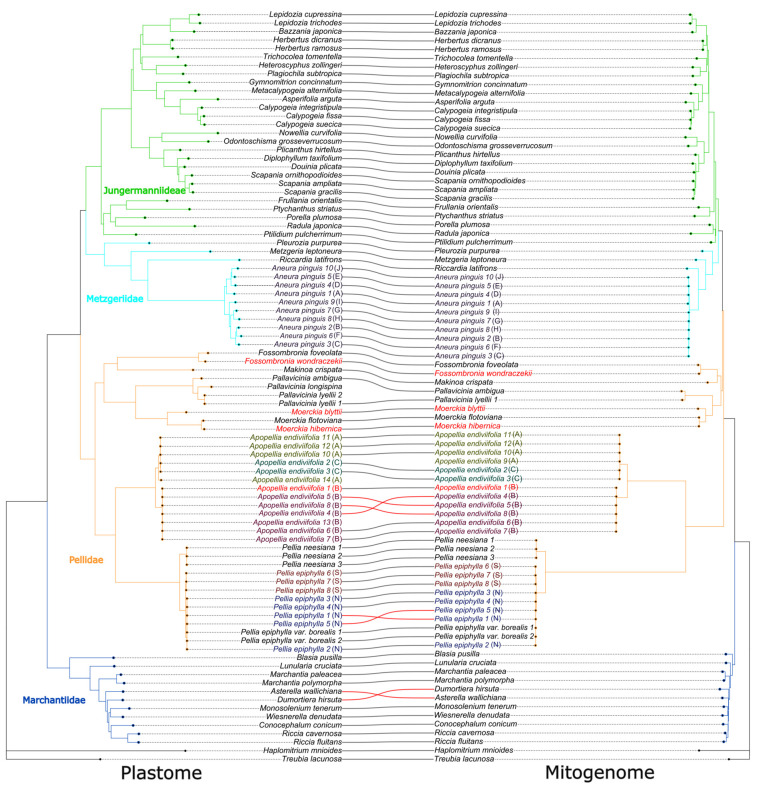
Phylogenetic topology inferred from concentrated plastid (**left**) and mitochondrial dataset (**right**) using ML method. Almost all clades are maximally supported; bootstrap values are provided in Supplementary Materials (Figures S1 and S2). Clade positions were optimized using cophylo function in the phylotools R package. Concentrated alignment for both datasets is present in Supplementary Materials (Supplementary Files S7 and S8).

Phylogenetic analyses based on complete organellar genomes revealed conflicting evolutionary topologies for the same group of plants. The primary difference in tree topology concerns the relationships among the three orders constituting the Pellidae: Pelliales, Fossombroniales, and Pallaviciniales. Data derived from complete chloroplast genomes indicate that the subclass Pellidae is a monophyletic group, where all three orders form a single, common evolutionary clade with high statistical support. Conversely, mitochondrial data analyses contradict the monophyly of this group, suggesting instead that the order Pelliales (represented by the genera *Pellia* and *Apopellia*) is the earliest diverging (basal) lineage within Jungermanniopsida. In this scenario, Pelliales does not form a common clade with Fossombroniales and Pallaviciniales but is rather a sister group to them and the remainder of the class (Figure 7).

**Figure 8:**
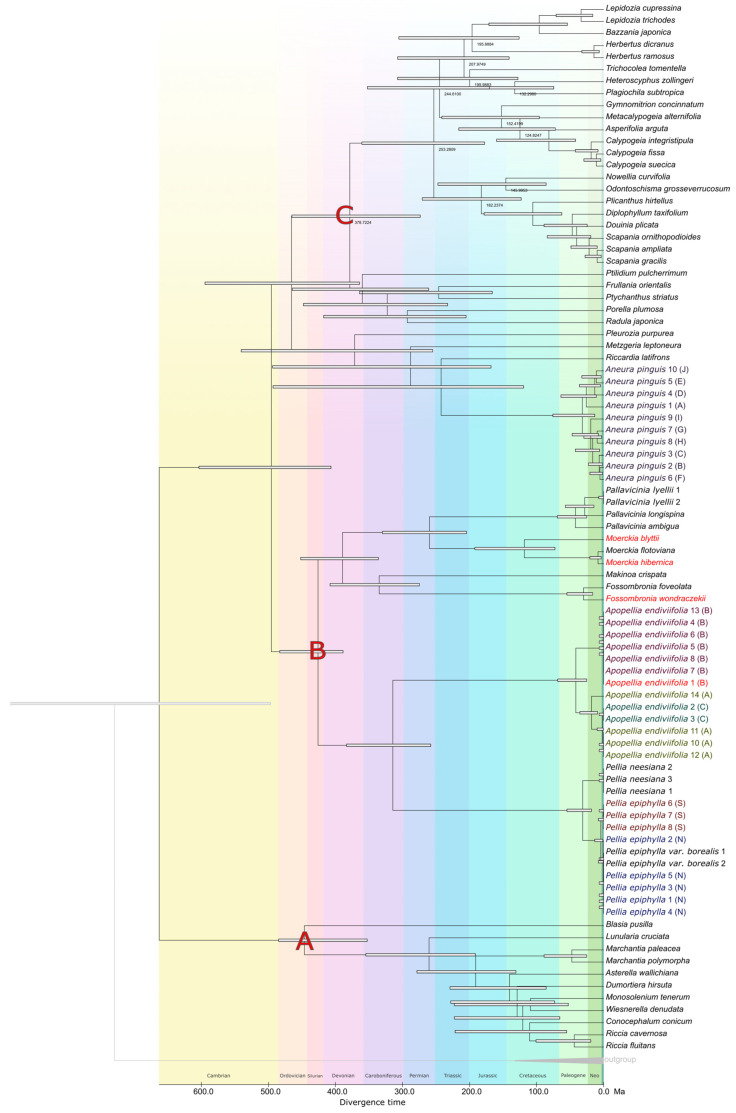
Timetree inferred by the RelTime method using plastome dataset. The RelTime analysis incorporated three calibration constraints ((**A**–**C**); Table S7) that were used to derive minimum and maximum bounds. Divergence time estimates are shown next to nodes in the tree and bars around each node represent 95% confidence intervals. Exact divergence times alongside node IDs are in Supplementary Materials (Figure S3); all timetree parameters are also provided in Table S5.

**Figure 9:**
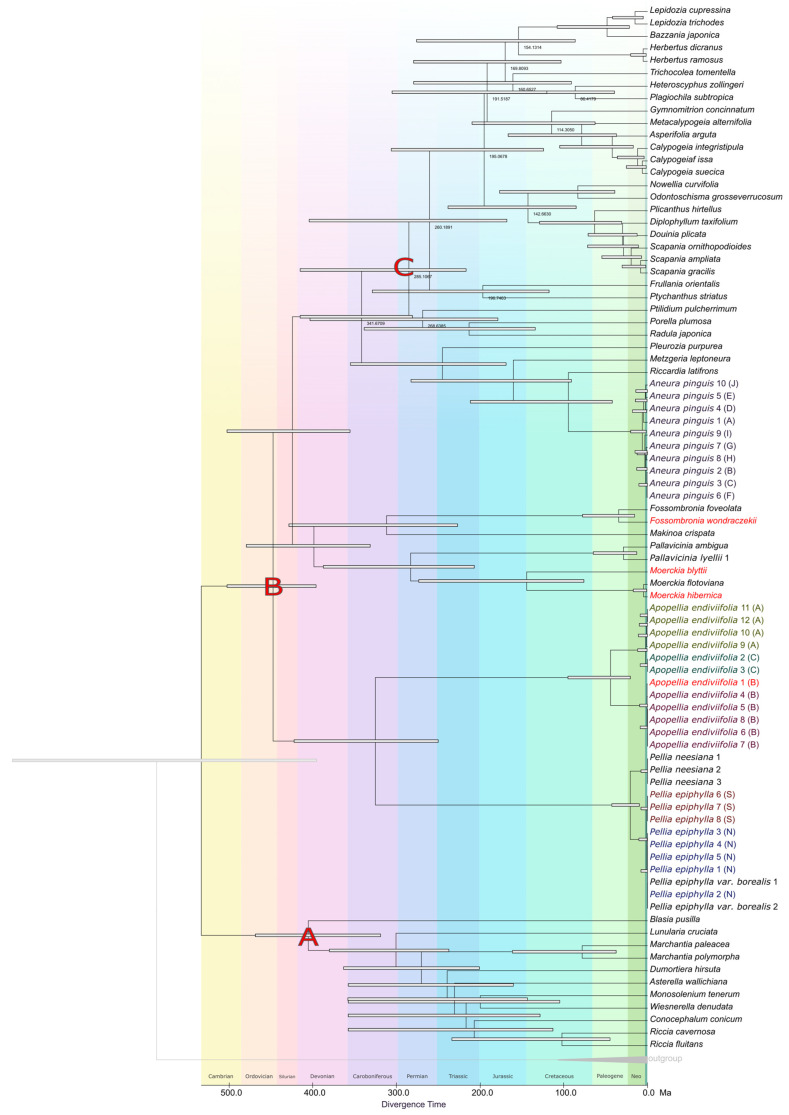
Timetree inferred by the RelTime method using mitogenome dataset. The RelTime analysis incorporated three calibration constraints ((**A**–**C**); Table S7) that were used to derive minimum and maximum bounds. Divergence time estimates are shown next to nodes in the tree and bars around each node represent 95% confidence intervals. Exact divergence times alongside node IDs are in Supplementary Materials (Figure S4); all timetree parameters are also provided in Table S6.

Time-calibrated phylogenies inferred from plastid (cp) and mitochondrial (mt) genomes recovered a shared deep-time framework for the lineages relevant to Pellidae, while also revealing pronounced cyto-organellar discordance in both topology and absolute node ages. In the cp chronogram, the deepest split across the analyzed sampling was dated to ~426.0 Ma, whereas the corresponding root in the mt chronogram was dated to ~447.3 Ma (Figure 8 and Figure 9, Tables S5 and S6). These estimates place the backbone divergences of the sampled Jungermanniopsida-related lineages in the Paleozoic and are compatible in magnitude with bryophyte-wide fossil-calibrated timetrees, despite substantial differences in marker systems and calibration density [37]. Within the Pellidae-relevant clades, several deep splits were dated consistently in the Carboniferous–Permian interval, but with systematic cp–mt offsets (Figure 8). The split separating the *Pellia* + *Apopellia* lineage from the remaining sampled lineages was dated to ~314.1 Ma in cp and ~341.2 Ma in mt, while the divergence of the *Moerckia* lineage from the *Fossombronia* + *Pallavicinia* clade was dated to ~259.5 Ma (cp) and ~283.2 Ma (mt) (Figure 8 and Figure 9, Tables S5 and S6).

The opposite direction of shift was inferred for *Makinoa crispata*, for which an older divergence was recovered in cp (~335.0 Ma) than in mt (~311.6 Ma). This indicates that cp–mt differences cannot be reduced to a single genome-wide tendency and are more consistent with lineage-dependent rate heterogeneity interacting with calibration constraints (Figure 8 and Figure 9, Tables S5 and S6).

At shallower taxonomic levels within Pelliales, a Neogene-scale split between *Pellia epiphylla* and *P. neesiana* was recovered in both timetrees, with divergence time estimates of ~30.9 Ma in the cp dataset and ~20.7 Ma in the mt dataset. The two main cryptic lineages within *P. epiphylla* [7,38] were inferred to have diverged between ~4.2 and ~1.3 Ma, and were thus substantially younger than the major lineages resolved within *Apopellia endiviifolia* [39], which diverged at ~41.4 Ma in cp and ~44.1 Ma in mt (Figure 8 and Figure 9, Tables S5 and S6). The close agreement between cp- and mt-derived divergence times in *Apopellia* was not unexpected, given that elevated mitochondrial evolutionary rates have been reported for this lineage, which may increase temporal resolution in mt-based dating within the genus [5].

The conflict between the chloroplast (cp) and mitochondrial (mt) DNA phylogenies mirrors the ongoing debate regarding the monophyly or paraphyly of the group Pellidae. Prior organellar studies on Pellidae have consistently revealed this incongruence: cp DNA analyses supported Pellidae as a monophyletic clade, whereas mt DNA analyses suggested its paraphyly, typically positioning Pelliales as the earliest diverging lineage within the class *Jungermanniopsida* [5,11]. Our current findings reproduce this exact topological discordance, indicating that the conflict between cp and mt DNA trees is a recurrent, systematic issue in the phylogenetic study of Pellidae, rather than an anomaly specific to our dataset.

A mechanistic explanation for why cp-based and mt-based inferences may disagree is provided by the broader liverwort literature, which indicates that the information content and evolutionary dynamics of organellar genomes differ markedly among compartments and lineages. Liverwort plastomes have been described as structurally conserved across the phylogeny, yet plastid genes have also been inferred to evolve unusually fast relative to nuclear genes in liverworts, with an estimated silent-substitution ratio of mitochondrial: plastid:nuclear ≈ 1:15:10, implying that the plastid signal can be particularly prone to saturation and model misspecification at deeper timescales [11]. Conversely, mitochondrial datasets can be affected by lineage-specific rate heterogeneity and by the complex interaction between genomic sequences and the extensive RNA editing known in Jungermanniopsida [9,40,41], which can introduce homoplasy if not adequately modeled [42,43,44]. Under such conditions, shifts in topology and node ages between cp and mt trees are expected to cluster around short internal branches and ancient rapid radiations—exactly the evolutionary setting in which Pellidae are frequently placed.

The question of whether Pellidae is a single group (monophyletic) or not (paraphyletic) also depends on how the nuclear DNA data is analyzed. A comprehensive molecular dating study of bryophytes [37], calibrations, concluded that Pellidae is “most likely paraphyletic” and noted that topological relationships shifted depending on whether raw nucleotide sequences or their translated amino acid sequences were analyzed. This observation is highly relevant to the present study, as our organellar inferences were also based on nucleotide data. Therefore, our findings—monophyly supported by chloroplast DNA and a basal position for Pelliales supported by mitochondrial DNA—represent hypotheses that might shift if analyzed using methods less sensitive to DNA sequence saturation (e.g., amino acid or codon-degenerated models) at deep evolutionary nodes. Another extensive phylogenomic study of liverworts [42] revealed substantial discordance among individual gene trees, as well as between nuclear and organellar datasets. They suggested this deep disagreement could be due to ancient hybridization events followed by incomplete lineage sorting (where ancestral variations persist). In summary, a cautious conclusion is that the ancient relationships of Pellidae-related groups are consistently found across different parts of the genome. However, whether Pellidae is monophyletic or paraphyletic, and the exact placement of Pelliales, will likely remain sensitive to which genome compartment is used (nuclear vs. organellar) and to the type of analysis (nucleotide vs. amino acid/codon-reduced). This suggests a combination of genuine biological complexity and analytical bias is at play [5,37,42].

## 3. Materials and Methods

A total of 90 chloroplast genomes and 86 mitochondrial genomes were included in the analyses. Of these, 86 chloroplast genomes and 82 mitochondrial genomes were retrieved from the NCBI database. Four chloroplast genomes lacked corresponding mitochondrial genome assemblies available in NCBI at the time of data collection. In this study, we newly generated and report four chloroplast genomes—*Apopellia endiviifolia* 1 (PX421529), *Fossombronia wondraczekii* (PX421530), *Moerckia blyttii* (PX421531), and *Moerckia hibernica* (PX421532)—together with four corresponding mitochondrial genomes derived from the same individual plants: *Apopellia endiviifolia* 1 (PX421533), *Fossombronia wondraczekii* (PX421534), *Moerckia blyttii* (PX421535), and *Moerckia hibernica* (PX421536). Accession numbers for all analyzed organellar genomes are provided in Supplementary Table S1. Letters after the number and taxonomic name of *Apopellia* (A, B, C), *Pellia* (N, S), and *Aneura* species (A–J) indicate different cryptic forms.

The analyzed specimen of *Apopellia endiviifolia* was included in the study due to the availability of corresponding full-length transcriptome sequencing data. Utilizing these data enabled the empirical validation and refinement of genomic annotations, allowing for the direct verification of the accuracy of gene structures identified within the newly sequenced organellar genomes.

Total genomic DNA was extracted from herbarium dried specimens using a Qiagen Plant mini spin kit (Qiagen, Hildenberg, Germany), with the exception of *Apopellia endiviifolia* B, which was extracted using an optimized HMW DNA extraction method [45] from in vitro material. The conditions of in vitro cultivation were the same as previously described for *Apopellia* [23]. The quality and quantity of extracted DNA was assessed using a Qubit BR DNA kit (Qiagen, Hildenberg, Germany) and a Cary4 spectrophotometer (Agilent, Santa Clara, CA, USA). The integrity and fragment distribution was analyzed using Genomic Tape on a Tapestation 4150 instrument (Agilent, Santa Clara, CA, USA).

The sequencing library was constructed using a Ligation Sequencing Kit Sqk-LSK.114 (ONT, Oxford, UK) in the case of *Apopelia* and a Native Barcoding Kit NBD-LSK.114 (ONT, Oxford, UK) for remaining specimens. The obtained libraries were quantified using a Qubit BR DNA kit and sequenced using R10.4.1 pores in Promethion2 (*Apopellia*) or Mk1C (rest of the samples) instruments (ONT, Oxford, UK). The obtained raw reads in POD5 format were basecalled using Dorado 1.0.1 (ONT, Oxford, UK) with an SUP model enabling modal accuracy at Q = 25.

Organellar genomes were assembled using Flye 2.9.1 [46] with minimum overlap set to 2000 bp and -m flag to enable unequal contig coverage. Obtained circular contigs were polished using Dorado 1.0.1 and annotated using Geneious Prime 2025.2.2.

### 3.1. Direct RNA Sequencing

Total RNA was extracted from land thalli of *Apopellia endiviifolia* of Lyna 1 line using the RNeasy Plant Mini Kit (Qiagen, Hilden, Germany), according to the manufacturer’s instructions. RNA concentration was measured using the Qubit RNA High-Sensitivity Assay Kit (Thermo Fisher Scientific, Waltham, MA, USA), and RNA integrity was assessed with the Agilent TapeStation system using High-Sensitivity RNA ScreenTape (Agilent, Santa Clara, CA, USA).

Direct RNA sequencing libraries were prepared from total RNA using the Oxford Nanopore Technologies (ONT, Oxford, UK) Direct RNA Sequencing Kit SQK-RNA004, following the manufacturer’s protocol. Prepared libraries were loaded onto R10.4.1 flow cells and sequenced on a PromethION 2 Solo platform until comparable read depth was obtained. Raw signal data (POD5 files) were basecalled using Dorado (v1.0; ONT, Oxford, UK) with the RNA004 superaccuracy model, generating basecalled reads for downstream analyses. Obtained reads were mapped onto organellar genomes using the RNA mode of native Geneious Prime 2025.2.2 with high sensitivity and novel intron discovery options.

For the purpose of this study, available, complete chloroplast and mitochondrial genomes were selected from the NCBI database, falling into the order of Pelliales, Pallaviciniales and Fossombroniales, as well as other taxa in the case of the dataset created for estimating divergence times in phylogenetic analysis. Datasets were enriched with genomes used in previous publications [5,11,21,22]. Annotation of all genomes was manually checked and corrected using Geneious Prime 2025.2.2.

### 3.2. Pi-Diversity (Nucleotide Diversity) Analysis

All genome alignments required for calculation of pi-diversity were created using MAFFT aligner in Geneious Prime 2025.2.2 with default parameters. Prepared alignments were loaded into the R environment, and pi-diversity values were obtained in sliding window analysis (window size = 500 bp, window jump = 100 bp) using PopGenome package for R [47] and visualized with ggplot2 [48]. Genes and sequence fragments with the highest pi-diversity (nucleotide diversity) values were highlighted.

### 3.3. Relative Synonymous Codon Usage (RSCU) Analysis

Each genome was concentrated into datasets containing only coding sequences (CDSs) for that genome using a custom Python 3.8 script which also respects CDS direction. Prepared genome sequences were loaded into the R environment; visualization and RSCU calculations were performed with the RSCUcaller 1.0 R package [49].

### 3.4. Phylogenetic Analysis

Using a series of custom-written Python 3.8 scripts, for each protein CDS (including introns), an alignment was created containing both CDSs and respective introns (if present) of a given gene for all cp/mt genomes. The final master alignments used for phylogenetic analyses comprised 87,514 bp for the plastid (cp) dataset and 96,230 bp for the mitochondrial (mt) dataset. Using these alignments, 2 master concentrated alignments were created using Python 3.8 script, one for cp genomes and one for mt genomes. Model search (for best-fit model), tree reconstruction and ultrafast bootstrap were all conducted with IQ-TREE [50]. To compute the support of phylogenetic groups in a maximum likelihood (ML)-based tree, an ultrafast bootstrap was performed using IQ-TREE with 1000 replicates. For mt alignment, the GTR+F+R3 model (General Time Reversible model with empirical base frequencies and FreeRate heterogeneity with three categories) was best-fit according to BIC; for cp alignment, the TVM+F+R6 model (Transversion Model with empirical base frequencies and FreeRate heterogeneity with six categories) was best-fit according to the same criteria. Obtained bootstrapped phylogenetic trees were loaded into MEGA software [51] in .nwk file format for divergence time estimation using the RelTime-Branch Lengths option. For both trees (mt/cp), 3 calibration points were used (fossil taxons: *Marchantites cyathodoides*, *Metzgeriothallus sharonae*, *Cheirorhiza brittae*), described in Bechteler et al. 2023 [37,52,53,54,55,56]. A branch containing *Haplomitrium mnioides* and *Treubia lacunosa* was set as an outgroup for both trees.

### 3.5. Inverted Repeat (IR) Boundary and Synteny Analysis

Determination of the boundaries of the inverted repeat (IR) regions were performed using Geneious Prime 2025.2.2. For each genome of Pellideae, the putative IRb region was extracted from the genome sequence. The extraction interval was extended to include substantial flanking sequences beyond the annotated 16S rRNA and 23S rRNA genes. The IRa region was extracted in this manner as a reverse complement. A pairwise alignment of the extracted IRb and reverse-complemented IRa sequences was constructed and inspected manually for the point of transition from high sequence identity (within the IR) to divergence, and mismatch (within the flanking parts of the sequence that spanned the fragment of the small single-copy region/large single-copy region), which made it possible to verify and precisely mark IR boundaries. To visualize the IR boundaries and the genes adjacent to the junction sites, a comparative map was generated using CPJSdraw [57]. Synteny analysis and visualization was performed using Mauve [58,59].

## 4. Conclusions

This study provides a comprehensive comparative analysis of the organellar genomes of the subclass Pellidae, significantly expanding the taxonomic sampling of this ancient liverwort lineage with eight newly sequenced genomes using Oxford Nanopore technology. Our findings challenge the traditional view of absolute structural stasis in liverwort organellar genomes, revealing a dynamic evolutionary history characterized by both extreme conservation and remarkable plasticity, as evidenced by the unique translocation of the *rps12–rps7–ndhB* gene cluster in Pallaviciniales and the radical miniaturization of the *Apopellia endiviifolia* mitogenome. The application of native RNA sequencing was crucial for refining organellar annotations, leading to the identification of previously unknown introns in the mitochondrial *atp1* gene and revealing the lowest intergenic region share (36.26%) recorded in the phylum to date. Furthermore, our analysis of nucleotide diversity and codon usage highlights divergent evolutionary dynamics between the two organellar compartments, where plastomes exhibit significantly higher variability and a more labile signal, while mitogenomes maintain greater structural and sequence stability. The observed robust topological conflict regarding the monophyly of Pellidae, with divergences dated to the Carboniferous–Permian interval, suggests that this discordance is a product of ancient, rapid radiations and lineage-specific rate heterogeneity rather than analytical artifacts. Ultimately, these results underscore that liverwort organellar evolution is a complex interplay of structural conservation and localized genomic shifts, demonstrating that a single-organelle approach is insufficient for resolving the backbone of early land plant evolution and highlighting the necessity for integrated, multi-compartment phylogenomic frameworks in future research.

## Data Availability

The chloroplast genome sequences generated and reported in this study have been deposited in the NCBI GenBank database under accession numbers PX421529 (*Apopellia endiviifolia* 1), PX421530 (*Fossombronia wondraczekii*), PX421531 (*Moerckia blyttii*), and PX421532 (*Moerckia hibernica*). The corresponding mitochondrial genome sequences derived from the same individual plants are available under accession numbers PX421533 (*Apopellia endiviifolia* 1), PX421534 (*Fossombronia wondraczekii*), PX421535 (*Moerckia blyttii*), and PX421536 (*Moerckia hibernica*). Additional organellar genomes analyzed in this study were retrieved from the NCBI GenBank database; a complete list of accession numbers is provided in Table S1. All other data supporting the findings of this study are available within the article and its Supplementary Materials.

## Supplementary Materials

The following supporting information can be downloaded at https://www.mdpi.com/article/10.3390/plants15070997/s1, Figure S1: Maximum-likelihood phylogenetic tree with bootstrap values for chloroplast dataset; Figure S2: Maximum-likelihood phylogenetic tree with bootstrap values for mitochondrial dataset; Figure S3: Phylogenetic tree with divergence times and node numbers (Node IDs) for chloroplast dataset; Figure S4: Phylogenetic tree with divergence times and node numbers (Node IDs) for mitochondrial dataset; Figure S5: RNA sequencing validation of the atp1 intron annotation in *Apopellia endiviifolia*. Direct RNA sequencing (DRS) reads were mapped to the plastid atp1 gene region to verify the presence and boundaries of the annotated introns. The upper panel shows read coverage across the atp1 locus, while the lower panel displays the alignment of individual RNA reads supporting the exon–intron structure; Supplementary File S1: Alignment in .fasta format containing chloroplast genomes of Fossombroniales, used in pi-diversity analysis; Supplementary File S2: Alignment in .fasta format containing mitochondrial genomes of Fossombroniales, used in pi-diversity analysis; Supplementary File S3: Alignment in .fasta format containing chloroplast genomes of Pallaviciniales, used in pi-diversity analysis; Supplementary File S4: Alignment in .fasta format containing mitochondrial genomes of Pallaviciniales, used in pi-diversity analysis; Supplementary File S5: Alignment in .fasta format containing chloroplast genomes of Pelliales, used in pi-diversity analysis; Supplementary File S6: Alignment in .fasta format containing mitochondrial genomes of Pelliales, used in pi-diversity analysis; Supplementary File S7: Concentrated alignment in .fasta format for chloroplast dataset used in phylogenetic analysis; Supplementary File S8: Concentrated alignment in .fasta format for mitochondrial dataset used in phylogenetic analysis; Table S1: List of genomes used in this study alongside their respective NCBI accession number and size; Table S2: Relative synonymous codon usage (RSCU) values for dataset consisting of chloroplast genomes; Table S3: Relative synonymous codon usage (RSCU) values for dataset consisting of mitochondrial genomes; Table S4: Pi-diversity values for middle point of each window in sliding window analysis; Table S5: Dated phylogenetic tree parameters for chloroplast dataset, divergence times (DivTime), relative evolutionary rates (Rate), and temporal confidence intervals (0.95; CI_Lower/CI_Upper) inferred using the RelTime method; Table S6: Dated phylogenetic tree parameters for mitochondrial dataset, divergence times (DivTime), relative evolutionary rates (Rate), and temporal confidence intervals (0.95; CI_Lower/CI_Upper) inferred using the RelTime method; Table S7: Overview on fossil calibrations used in this study; Table S8. Average sequencing coverage depth for Nanopore assemblies of analyzed plastid genomes.
