# Supplementary figures and images for: Comparative Organellar Genomics of Pellidae: Insights into Codon Usage, Nucleotide Diversity, and Structural Evolution
Source: Plants (Basel). 2026 Mar 24;15(7):997. doi: 10.3390/plants15070997 (PMC13074886; doi:10.3390/plants15070997)

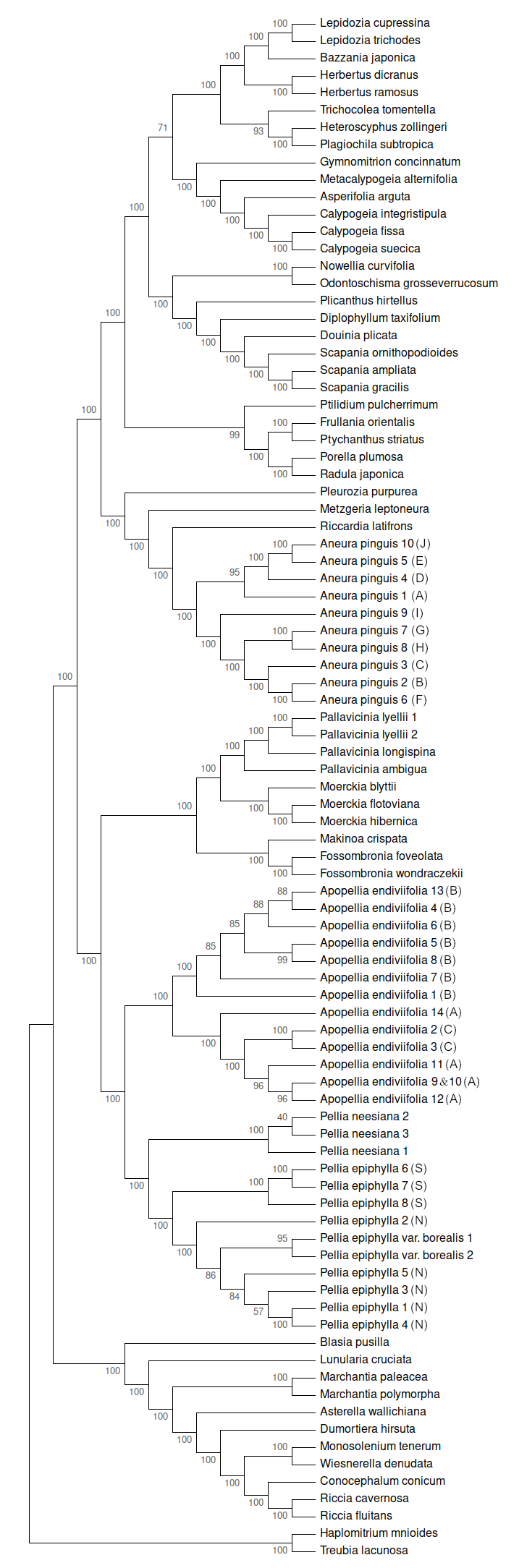

Supplement: Supplementary file 1 [file plants-15-00997-s001.zip › Figure S1.png]

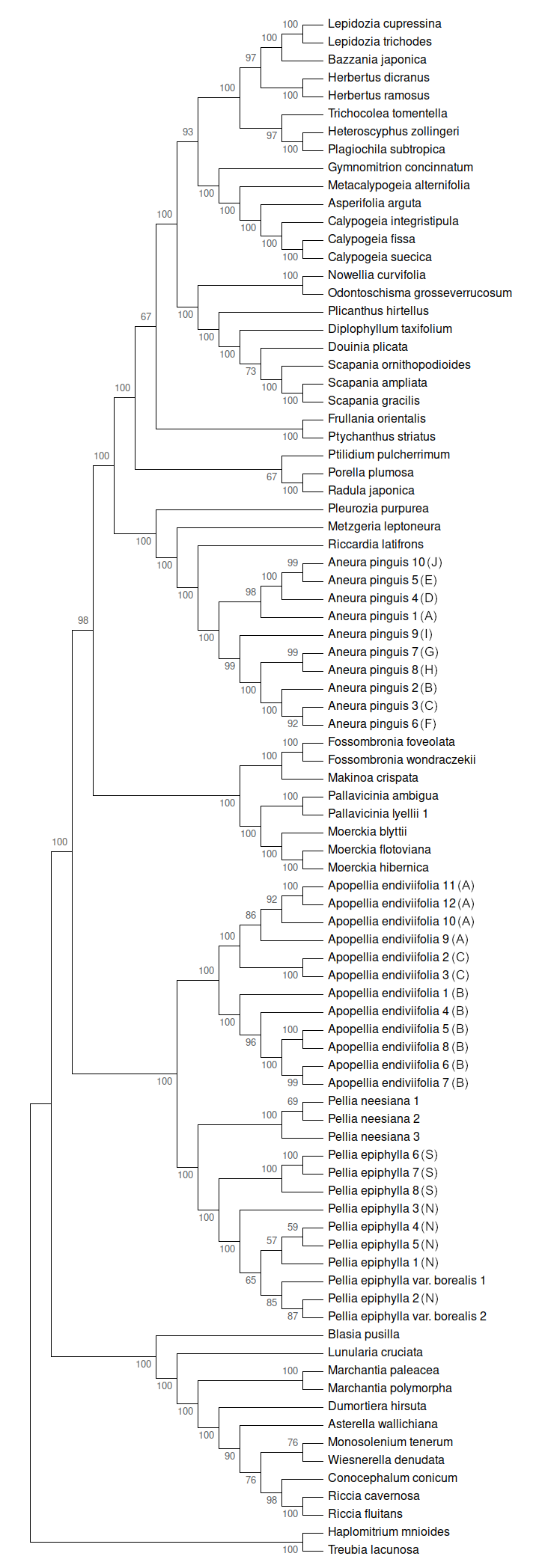

Supplement: Supplementary file 1 [file plants-15-00997-s001.zip › Figure S2.png]

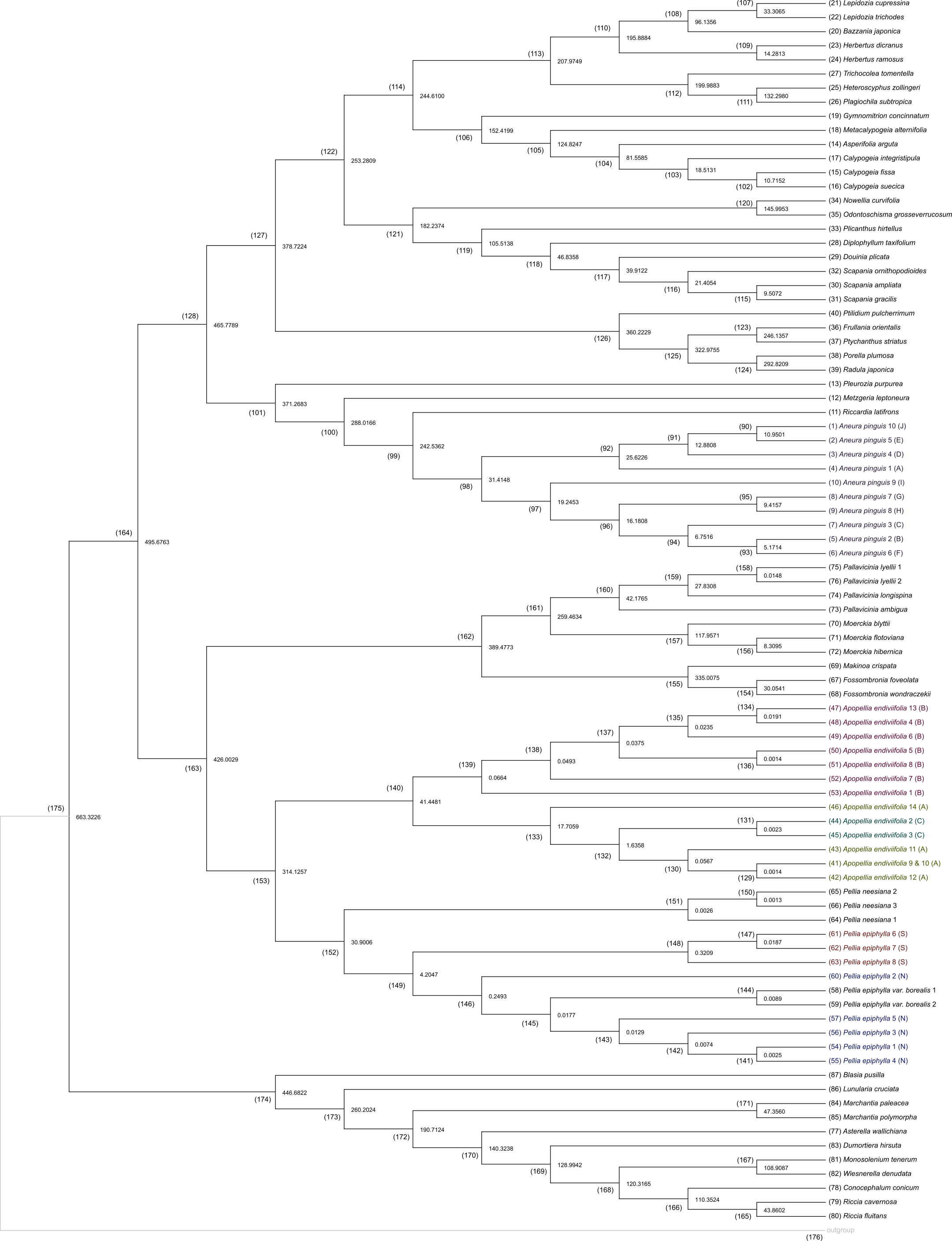

Supplement: Supplementary file 1 [file plants-15-00997-s001.zip › Figure S3.png]

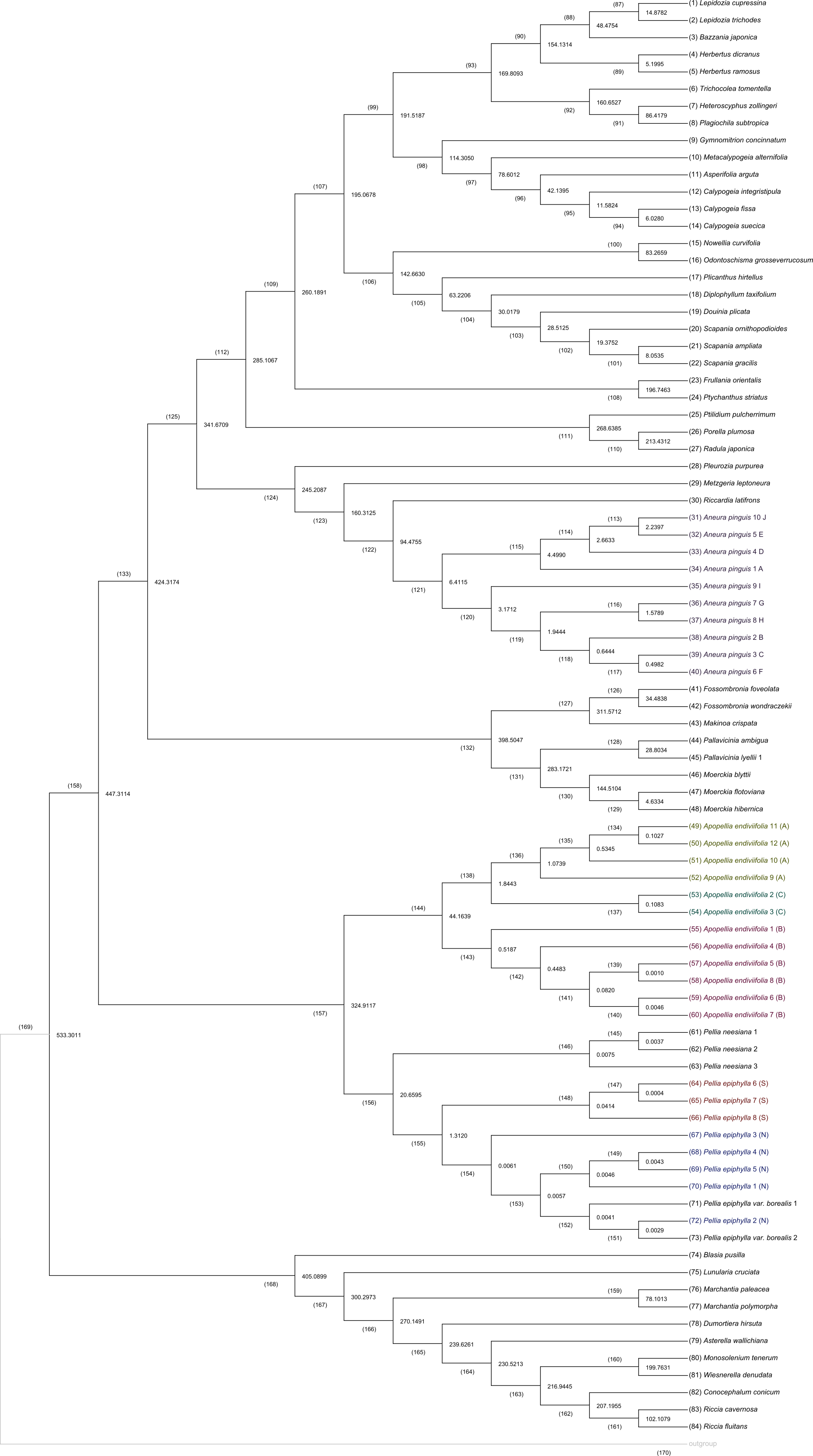

Supplement: Supplementary file 1 [file plants-15-00997-s001.zip › Figure S4.png]

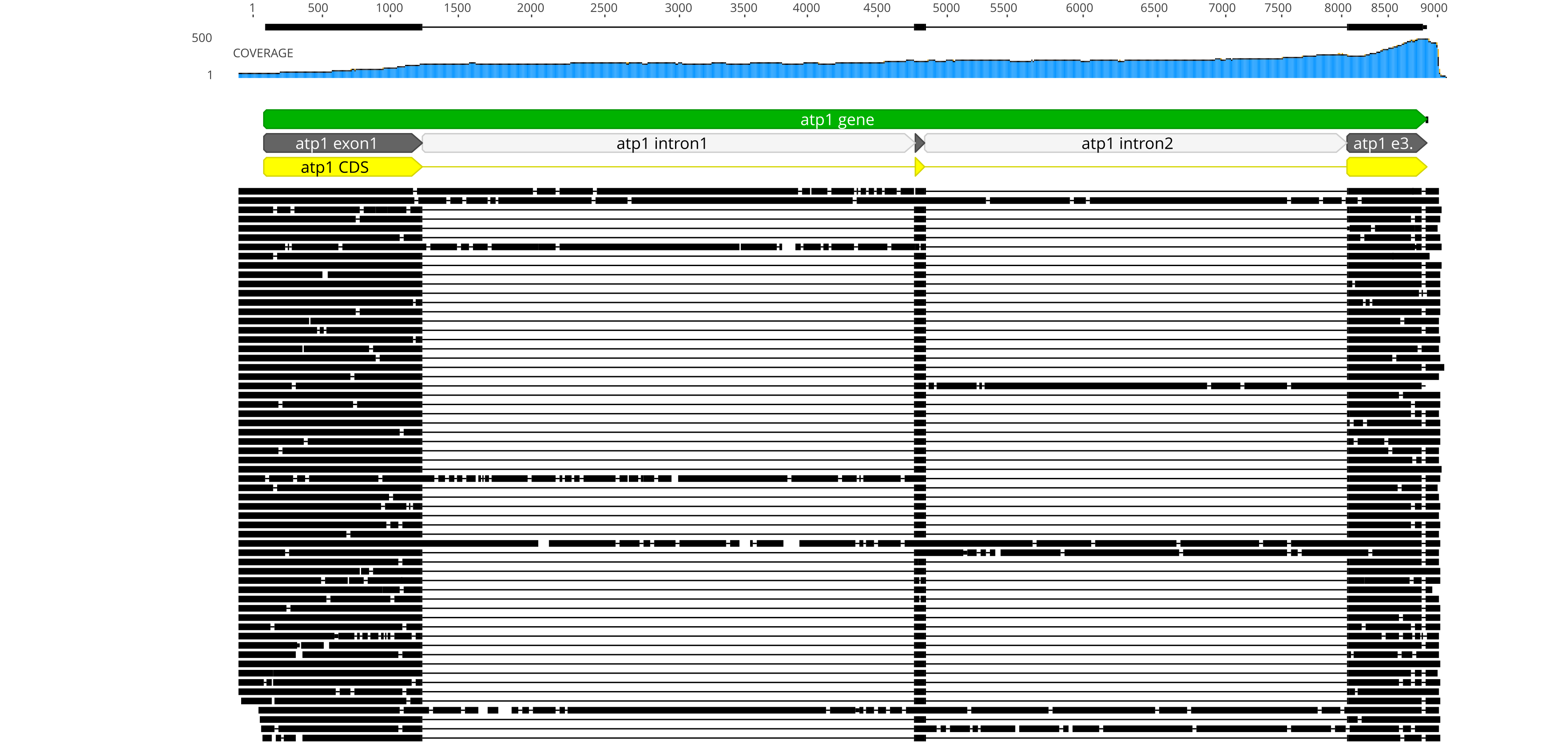

Supplement: Supplementary file 1 [file plants-15-00997-s001.zip › Figure S5.png]
